# Irreducible Inguinal Bladder Herniation Containing an Intravesical Calculus: A Rare Dual Pathology

**DOI:** 10.7759/cureus.101894

**Published:** 2026-01-20

**Authors:** Rahul A Mishra, Dinesh Ramaswamy, Shanmugasundaram Rajaian

**Affiliations:** 1 General Surgery, Grant Government Medical College, Mumbai, IND; 2 Surgical Gastroenterology, SRM Institute of Medical Sciences, Chennai, IND; 3 Urology, SRM Institute of Medical Sciences, Chennai, IND

**Keywords:** cystoscopic vesicolitholapaxy, inguinal bladder hernia, open mesh hernioplasty, sliding hernia, surgical case report, urinary bladder hernia, vesical calculus within inguinal hernia

## Abstract

Inguinal bladder herniation is a rare clinical condition, and its association with a vesical calculus within the herniated bladder segment is exceptionally uncommon. We report the case of a 68-year-old man who presented with bilateral inguinal herniae accompanied by hematuria. He was evaluated with urine culture and sensitivity testing, blood investigations, and computed tomography (CT). CT imaging demonstrated a left inguinal hernia containing a portion of the urinary bladder with an associated 2.5 × 1.8 cm vesical calculus. In collaboration with the urology team, the patient underwent bilateral open mesh hernioplasty with vesicolitholapaxy using a holmium:YAG (Ho:YAG) laser. Successful reduction of the herniated bladder segment and complete removal of the calculus were achieved. Early preoperative identification of bladder herniation with an associated stone through imaging played a critical role in surgical planning and helped avoid inadvertent bladder injury during surgery. This case underscores the importance of considering bladder herniation in elderly male patients presenting with inguinal herniae and urinary symptoms, as timely diagnosis and coordinated surgical-urological management can optimize outcomes and reduce perioperative risk.

## Introduction

Inguinal hernia is a common surgical condition; however, herniation of the urinary bladder is rare, occurring in approximately 1%-4% of all inguinal herniae and in up to 10% of men over 50 years of age [1,2]. Most bladder herniae are asymptomatic and are often discovered incidentally during imaging studies or surgical procedures [3,4]. Elderly obese males with bladder outlet obstruction are particularly predisposed due to chronic straining, progressive weakening of the pelvic musculature, and increased intravesical pressure [5,6].

Herniation of the bladder is clinically significant because it may present subtly with nonspecific urinary symptoms, such as hematuria, dysuria, or the characteristic two-stage micturition phenomenon. More importantly, unrecognized bladder involvement places patients at substantial risk of inadvertent bladder injury during hernia repair, potentially leading to significant postoperative morbidity.

The coexistence of a vesical calculus within a herniated portion of the bladder is exceedingly rare and typically reflects chronic urinary stasis and long-standing obstruction [7-9]. When the bladder herniates into the inguinal canal, the dependent herniated segment retains urine, facilitating stone formation and increasing the risk of complications such as incarceration, infection, or obstruction [10]. Accurate preoperative identification using cross-sectional imaging is therefore essential for safe surgical management.

The purpose of this case report is to describe a rare presentation of inguinal bladder herniation with an intravesical calculus presenting with inguinal swelling and hematuria; to highlight the diagnostic role of computed tomography (CT) in identifying associated bladder pathology, such as calculi or tumors, prior to inguinal hernia repair; and to emphasize the importance of multidisciplinary surgical and urological management in preventing iatrogenic injury and optimizing patient outcomes.

## Case presentation

**Figure 1:**
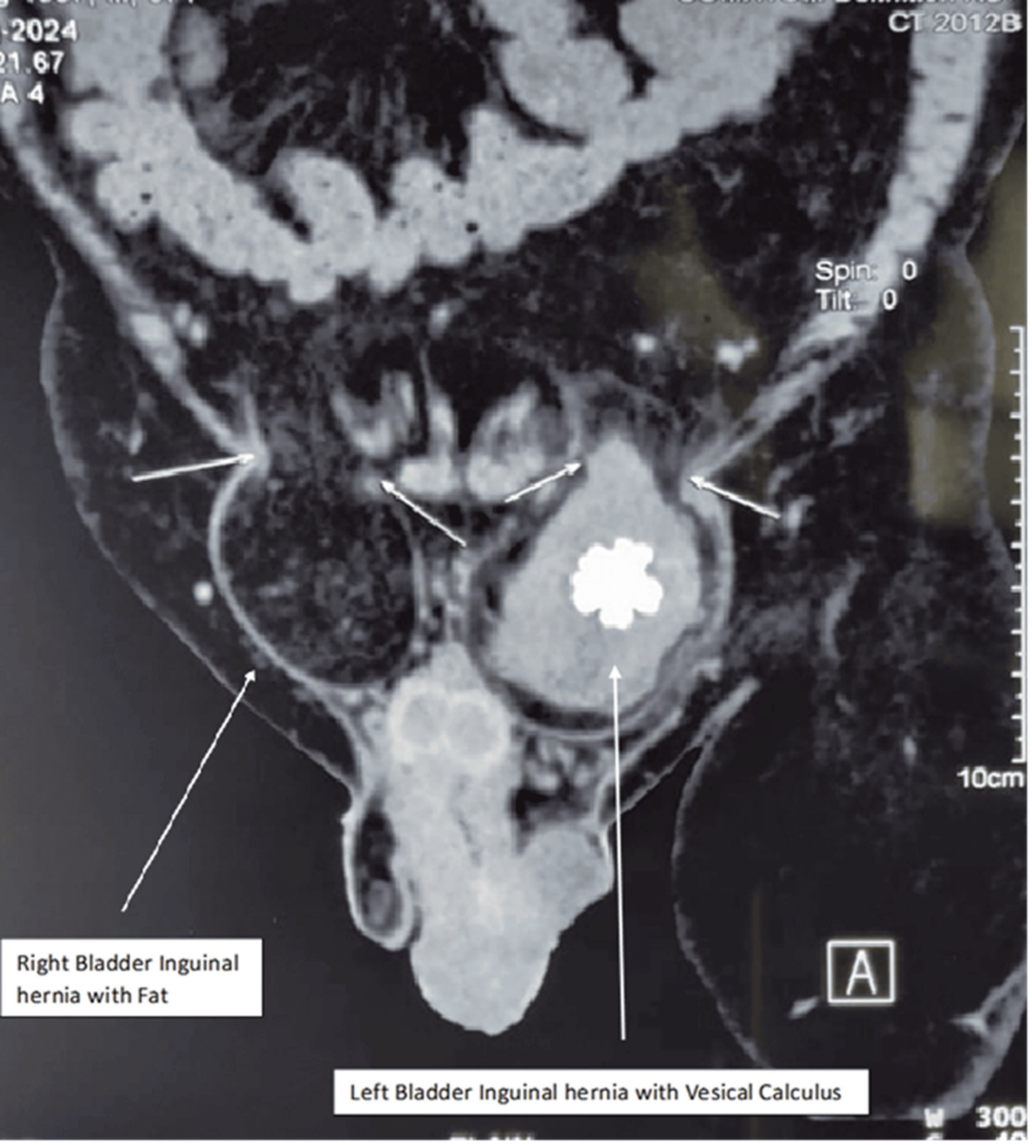
Computed tomography showing urinary bladder inguinal hernia with vesical calculus on the left side

**Figure 2:**
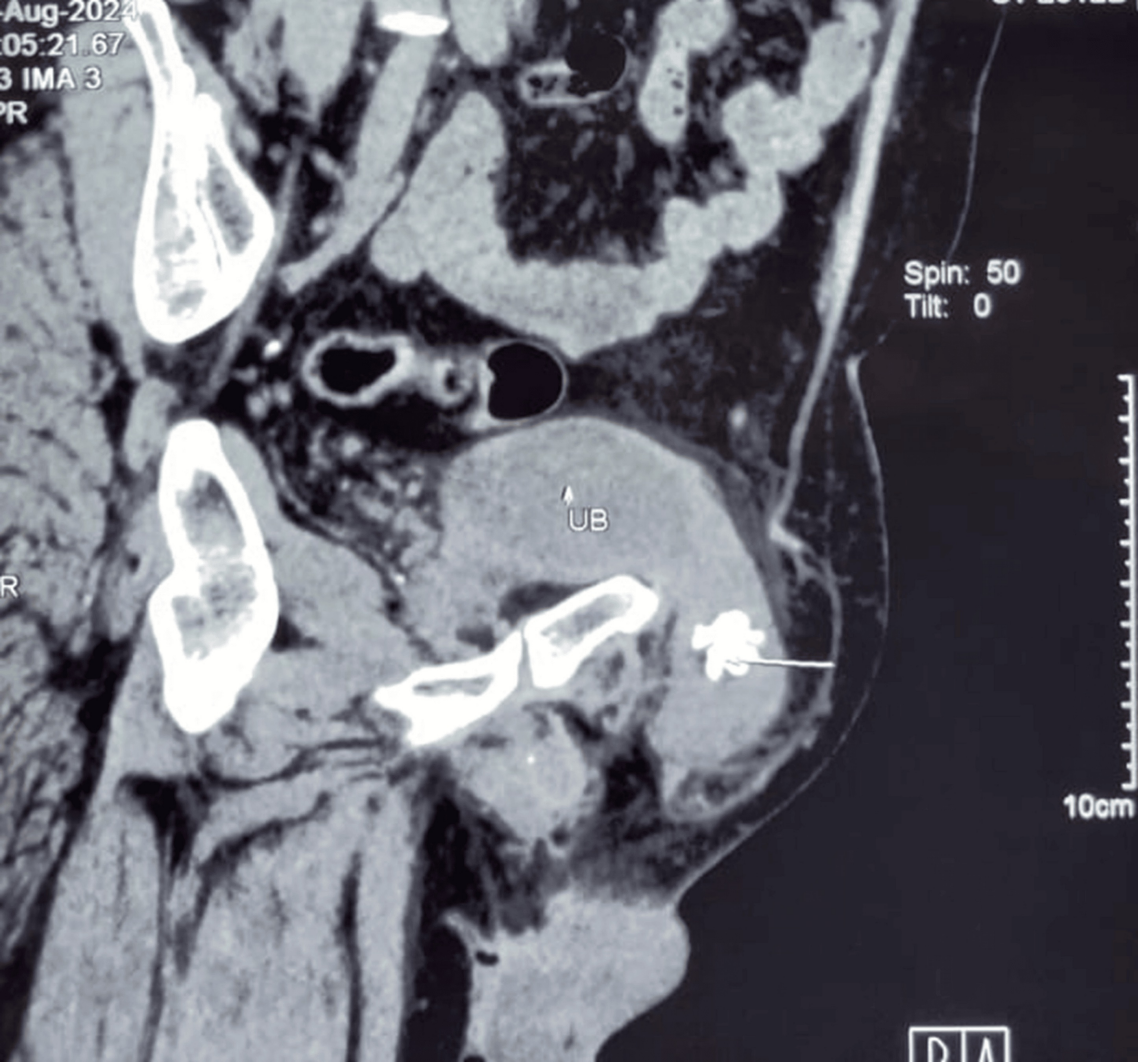
Computed tomography showing herniation of the urinary bladder into the left inguinal canal with an intravesical calculus (small white arrow)

A 68-year-old male presented with a five-year history of left-sided groin swelling, which had become irreducible over the past six months, consistent with an incarcerated inguinal hernia. He later noticed a right inguinal swelling two months prior to presentation and had a history of intermittent gross hematuria and difficulty in passing urine. On evaluation at our center, clinical examination revealed bilateral inguinal herniae, with the left side larger than the right. The left inguinal hernia was irreducible, while the right was completely reducible. Clinically, the presence of the urinary bladder and calculus within the hernia was not suspected, and the diagnosis was established only after a CT of the abdomen and pelvis was performed for the evaluation of hematuria. CT imaging demonstrated a left inguinal hernia containing a portion of the urinary bladder with a spiculated radio-opaque calculus measuring 2.5 × 1.8 cm within the herniated bladder segment (Figures 1, 2). Associated bladder wall thickening with features suggestive of cystitis was also noted. The right inguinal hernia contained only fat. Urine culture and sensitivity testing were sterile. A combined surgical and urological approach was planned for management.

**Figure 3:**
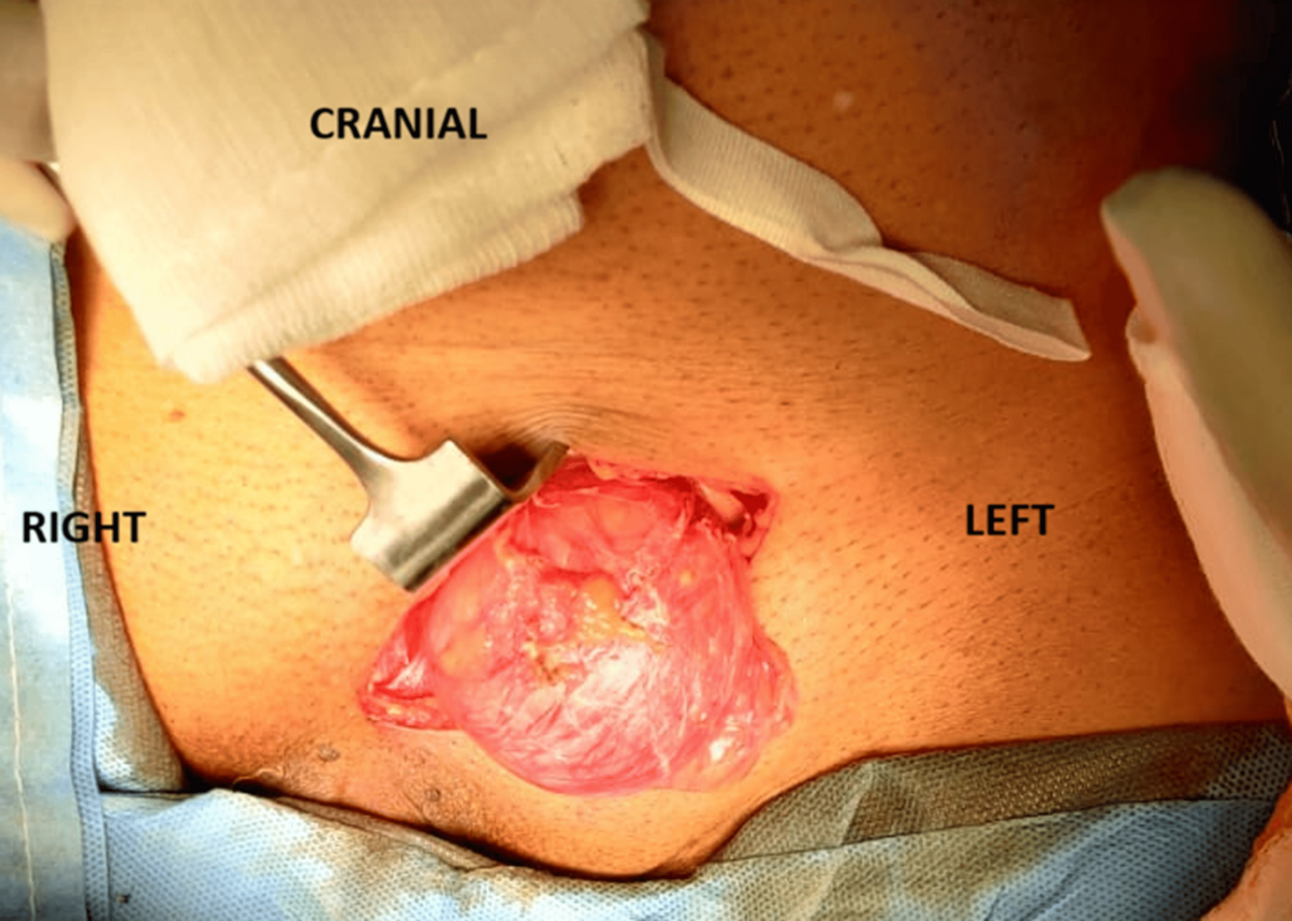
Intraoperative image of the left inguinal hernia with bladder as content

**Figure 4:**
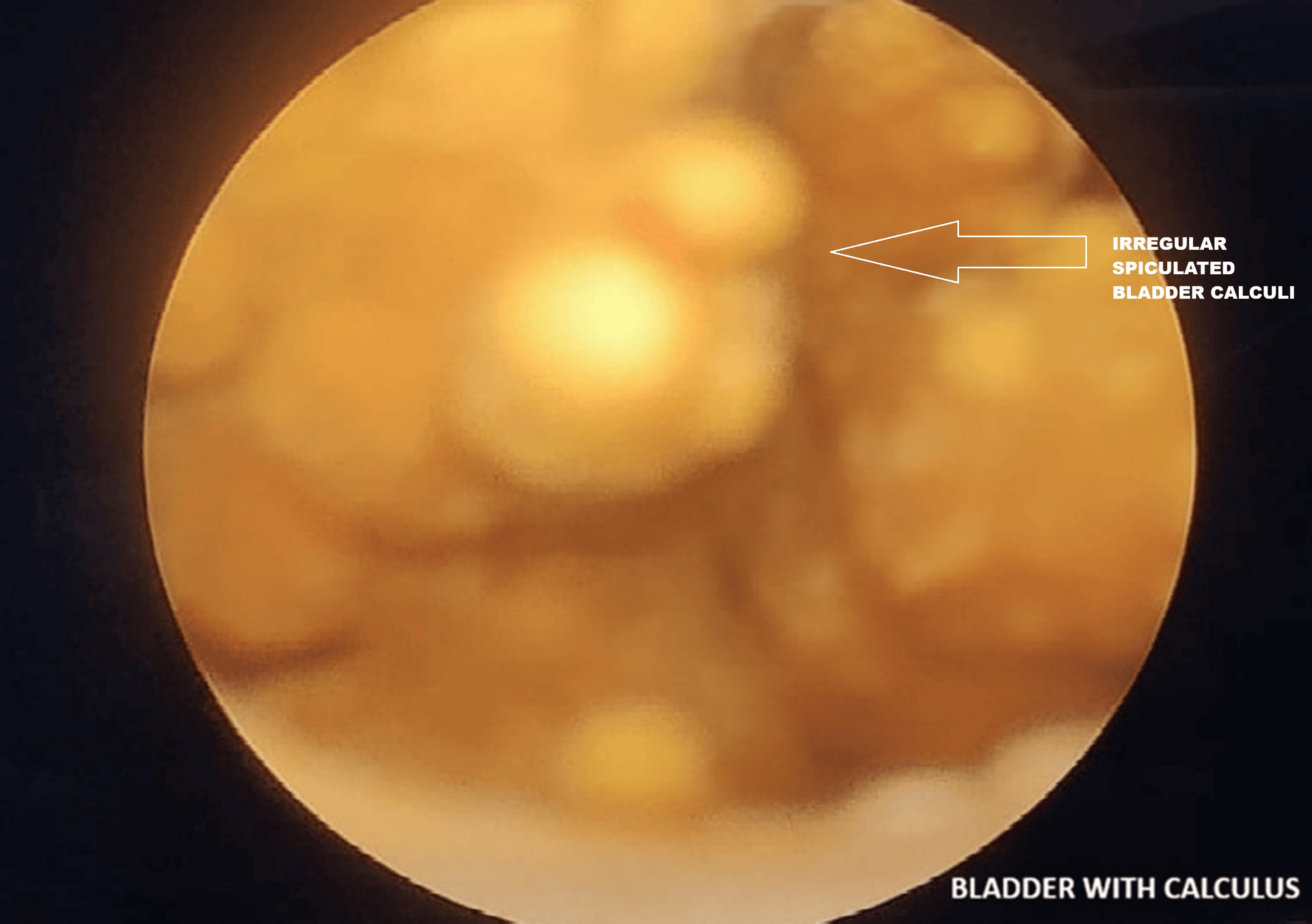
Cystoscopy showing irregular spiculated bladder calculus

After completing the preanesthetic evaluation and obtaining fitness for surgery, the patient was taken up for operative management. Initial cystoscopy was performed, which showed normal bladder mucosa and a small, non-occlusive prostate; however, the calculus was not visualized at that time. The surgical team then proceeded with an open approach and attempted reduction of the left inguinal hernia. Reduction was unsuccessful due to the presence of the calculus within the herniated bladder segment (Figure 3). With external manipulation and compression, a large irregular spiculated calculus measuring approximately 3 cm was displaced back into the bladder lumen and subsequently identified, likely causing abrasion of the bladder mucosa (Figure 4). Vesicolitholapaxy was performed using a holmium:YAG (Ho:YAG) laser. The bladder stone was successfully fragmented and evacuated with irrigation. Following stone removal, the left-sided incarcerated bladder segment was completely reduced. A three-way Foley catheter was placed for postoperative bladder irrigation and monitoring.

Bilateral open inguinal hernia repair was performed using the standard Lichtenstein tension-free mesh technique. Intraoperative findings revealed a left-sided direct inguinal hernia containing the urinary bladder. After complete reduction of the herniated bladder segment, posterior wall repair was performed using Prolene sutures, followed by placement of a 7.6 × 15 cm Prolene mesh. On the right side, a direct inguinal hernia was identified and repaired in a similar manner with mesh reinforcement.

Postoperatively, the patient remained stable and had an uneventful recovery. He was voiding well at the one-week follow-up. At the three-month follow-up, there was no recurrence of symptoms or clinical signs.

## Discussion

Urinary bladder herniation into the inguinal canal is an uncommon clinical entity and is often underdiagnosed due to its nonspecific presentation [1,5]. Most cases occur in elderly males and are associated with chronic bladder outlet obstruction, obesity, prostatic enlargement, and weakening of the pelvic musculature [2]. Patients may present with inguinal swelling, lower urinary tract symptoms, hematuria, or the characteristic two-stage micturition phenomenon; however, many cases remain asymptomatic [6].

The presence of a vesical calculus within a herniated bladder segment is particularly rare and typically reflects chronic urinary stasis [3,7]. Chronic outlet obstruction, along with the dependent position of the herniated bladder segment, promotes urine retention, progressive trabeculation, and stone formation [4]. When such hernias become incarcerated, the risk of ischemia, infection, and inadvertent bladder injury during hernia repair increases significantly, especially if the diagnosis is not established preoperatively [7].

Comparison with previously reported cases

Several previously reported cases in the literature have described bladder herniation associated with vesical calculi; however, most of them involved smaller calculi or reducible hernias. Liu et al. reported a bladder herniation containing a calculus but without significant incarceration or severe inflammatory changes [3]. Maheshwari et al. documented multiple vesical calculi in a large incarcerated hernia, but the stone morphology differed from the spiculated calculus seen in our case [7]. Duran et al. presented a scrotal bladder herniation containing a calculus but without associated bladder wall thickening or cystitis [10].

In contrast, our case is unique because of the spiculated 2.5 × 1.8 cm calculus that caused true incarceration of the herniated bladder segment, resulting in bladder wall thickening and cystitis. The combination of stone size, morphology, and the degree of incarceration makes this case comparatively rare and clinically relevant.

CT is considered the imaging modality of choice, as it reliably delineates the herniated bladder segment, identifies associated calculi, and evaluates bladder wall changes [2,8]. Early radiologic diagnosis significantly reduces the likelihood of intraoperative bladder injury.

Differential diagnosis

The differential diagnosis for groin swelling associated with urinary symptoms includes inguinal bladder hernia, ureterocele herniation, herniated bladder diverticulum, large hydrocele with mass effect, femoral hernia compressing the bladder neck, and inguinoscrotal hernias containing bowel or pelvic fat. Rarely, inguinal hernias may involve ureteric structures, leading to urinary complaints. In such cases, the presence of hematuria, dysuria, two-stage micturition, or acute urinary retention should raise clinical suspicion for bladder involvement, warranting cross-sectional imaging for accurate preoperative assessment.

Management involves careful reduction of the herniated bladder, addressing associated conditions such as calculus removal, and performing tension-free mesh hernioplasty. Mesh repair is considered safe when bladder integrity is preserved and there is no active infection [6,9]. As demonstrated in this case, a combined urological and surgical approach can result in favorable outcomes [7,9].

## Conclusions

Inguinal bladder herniation with a vesical calculus is an uncommon but clinically significant condition that requires a high index of suspicion, particularly in elderly males presenting with groin swelling and urinary symptoms. Early cross-sectional imaging is essential for identifying bladder involvement and guiding safe surgical planning. A combined urological and surgical approach enables complete management of both the hernia and associated pathology. Prompt recognition and appropriate intervention can prevent bladder injury and optimize patient outcomes.
